# Supplementary material for: Cellular conditions of weakly chelated magnesium ions strongly promote RNA stability and catalysis
Source: Nat Commun. 2018 Jun 1;9:2149. doi: 10.1038/s41467-018-04415-1 (PMC5984629; doi:10.1038/s41467-018-04415-1)
Supplement: Supplementary file 1 — Supplementary Information [file 41467_2018_4415_MOESM1_ESM.pdf]

Supplementary Information

Cellular conditions of weakly chelated magnesium ions strongly promote RNA stability and catalysis

Ryota Yamagami[1,2], Jamie L. Bingaman[1,2], Erica A. Frankel[1,2] and Philip C. Bevilacqua*[,1,2,3]

Author affiliations:

[1]Department of Chemistry, Pennsylvania State University, University Park 16802, Pennsylvania, USA.

[2]Center for RNA Molecular Biology, Pennsylvania State University, University Park 16802, Pennsylvania, USA.

[3]Department of Biochemistry and Molecular Biology, Pennsylvania State University, University Park 16802, Pennsylvania, USA.

***Corresponding author:**

Philip C. Bevilacqua

Phone: +1-814-863-3812

Fax: +1-814-865-2927

E-mail: pcb5@psu.edu

Table of Contents

1. Supplementary Figures

Supplementary Figure 1. Magnesium ion and amino acid is a major metal ion and metabolite in *Escherichia coli* cells.................... S4

Supplementary Figure 2. Structures and proposed enzymatic mechanism of HDV-like ribozyme and CPEB3 ribozyme studied in this work S5

Supplementary Figure 3. Glutamate does not affect RNA folding and stability S6

Supplementary Figure 4. Enzymatic activity of the *glmS* ribozyme is stimulated in both bacterial and eukaryotic cell mimic conditions.................... S7

Supplementary Figure 5. Enzymatic activity of the hammerhead ribozyme 16 is stimulated by weakly bound magnesium.................... S9

Supplementary Figure 6. Glutamate-chelated magnesium contributes on RNA compaction.................... S11

Supplementary Figure 7. *glmS* ribozyme shows similar thio effect in 0.5 mM glutamate-chelated magnesium condition to 3.6 mM Mg^{2+}_{free}.................... S12

Supplementary Figure 8. Weakly chelated magnesium shows similar free-activation energy to unbound magnesium S14

Supplementary Figure 9. Crowding conditions with chelated magnesium show greater amplitudes for RNA catalysis S15

3. Supplementary Tables

Supplementary Table 1. Estimated concentrations of cellular amino acids and magnesium complexes in *Escherichia coli* S16

Supplementary Table 2. All melting profiles.................... S17

Supplementary Table 3. Kinetics parameters of CPEB3 ribozyme for self-cleaving reaction in several conditions.................... S18

Supplementary Table 4. Kinetics parameters of *glmS* ribozyme for self-cleaving reaction in several conditions S19

Supplementary Table 5. Kinetics parameters of hammerhead 16 for self-cleaving reaction in several conditions S19

Supplementary Table 6. SAXS determined R_g, D_{max} and molecular weight in various conditions S20

Supplementary Table 7. Thio effect for self-cleaving reaction in several conditions . S20

4. References S21

Supplementary Figures

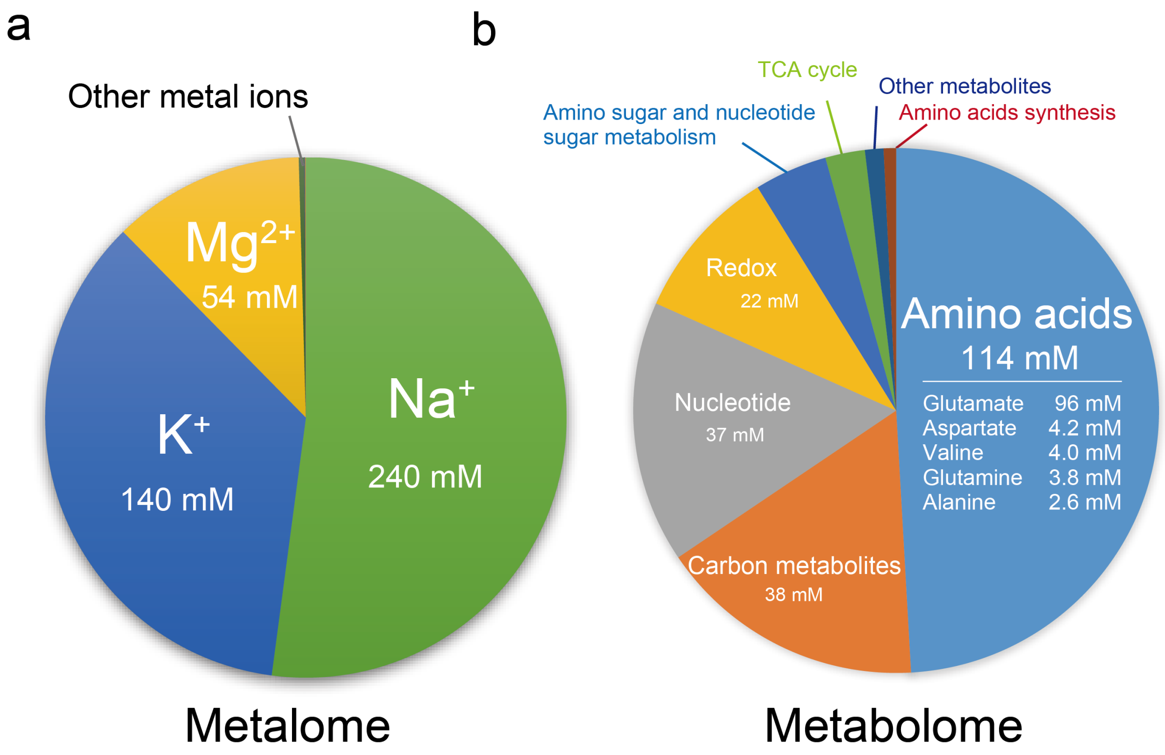

Supplementary Fig. 1 | Magnesium ions and amino acids are major metal ion and metabolite in *Escherichia coli* cells. (a) Pie chart of metalome analysis. Dataset was obtained from [1]. Concentrations are shown as total concentrations. (b) Pie chart of metabolome analysis. Dataset was obtained from [2,3]. Amino acids occupy approximately half of all metabolites.

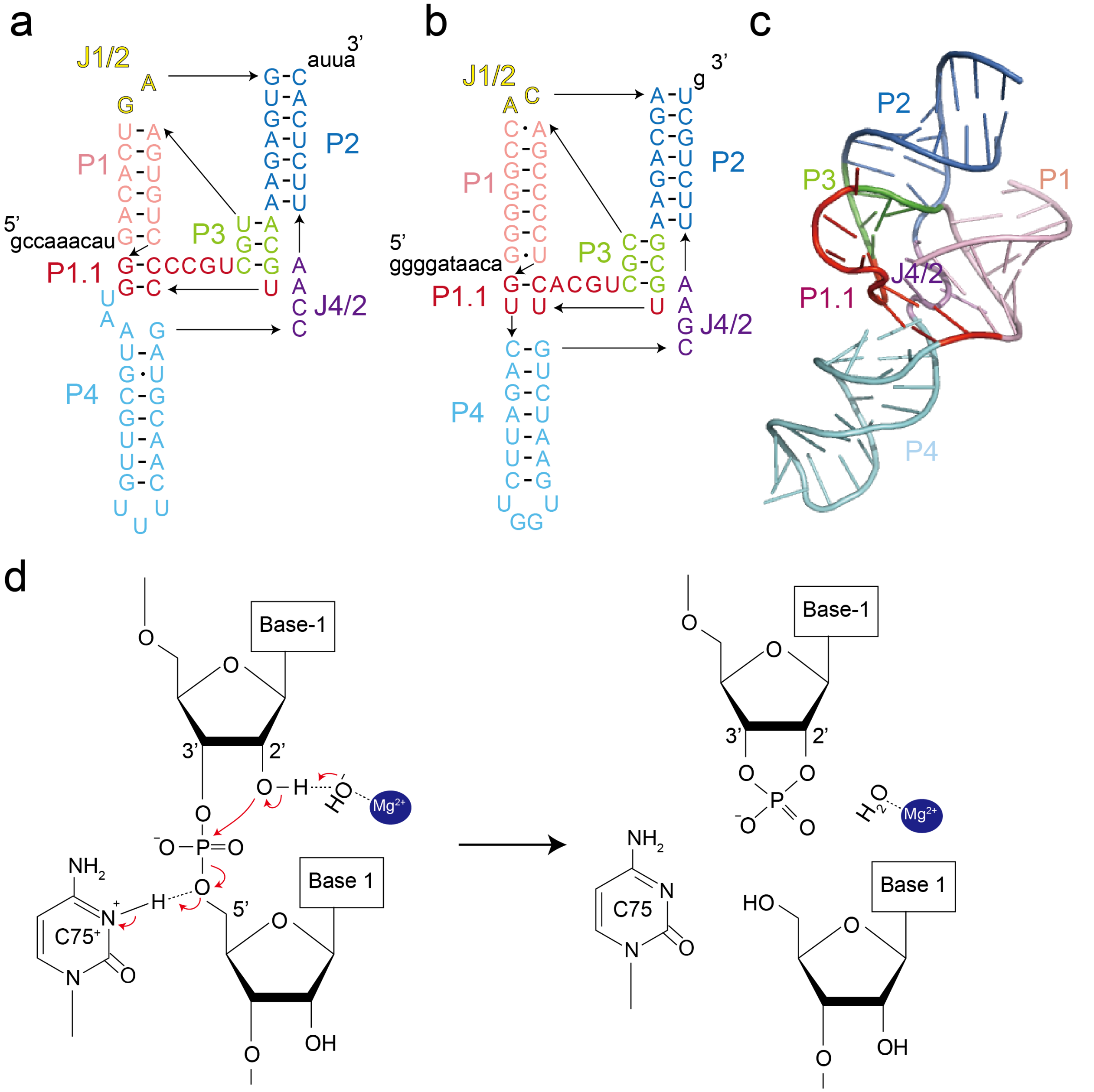

Supplementary Fig. 2 | Structures and proposed enzymatic mechanism of HDV-like ribozyme and CPEB3 ribozyme studied in this work. (**a**) The primary sequence and secondary structure of drz-spur-3. (**b**) The primary sequence and secondary structure of CPEB3 ribozyme. (**c**) The three-dimensional structure of HDV ribozyme (PDB:3NKB) illustrated by PyMOL. Each stem and loop region is colored with same as (**a**) and (**b**). (**d**) Catalytic mechanism of HDV-like ribozyme. The nucleophilic 2'-hydroxyl group is activated by an essential hydrated magnesium, located in the catalytic cavity, and the leaving group is stabilized by protonated $C75^{+}$.

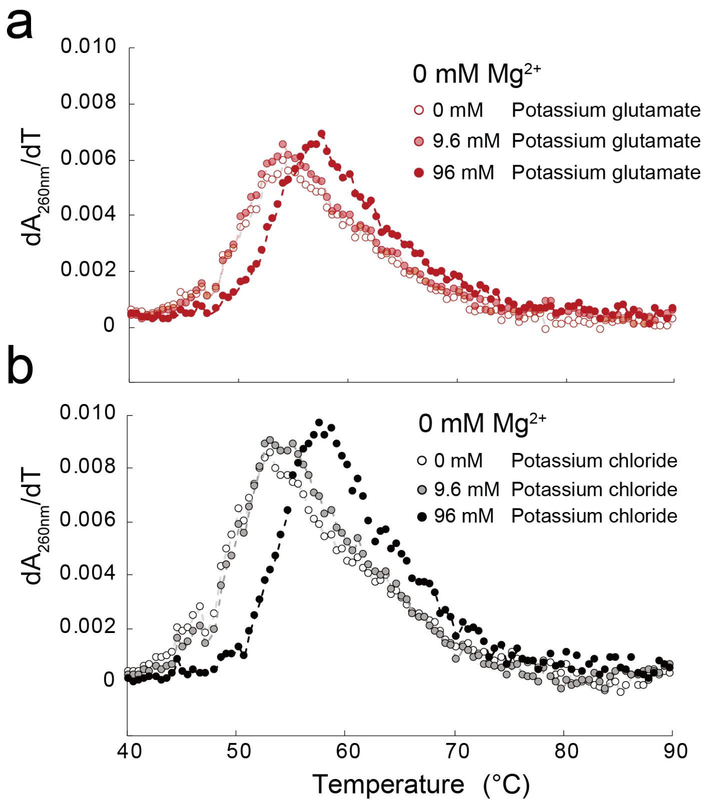

Supplementary Fig. 3 | Glutamate does not affect RNA folding and stability. Thermal denaturation experiments conducted using cleaved drz-spur-3 ribozyme in a background of 140 mM KCl/0 mM Mg^{2+}. (a) Unfolding curves in the presence of potassium glutamate. 0 mM (open circle), 9.6 mM (light red) and 96 mM (red) potassium glutamate were tested. (b) Unfolding curves in the presence of additional KCl. 0 mM (open circle), 9.6 mM (grey) and 96 mM (black) additional potassium chloride conditions were tested.

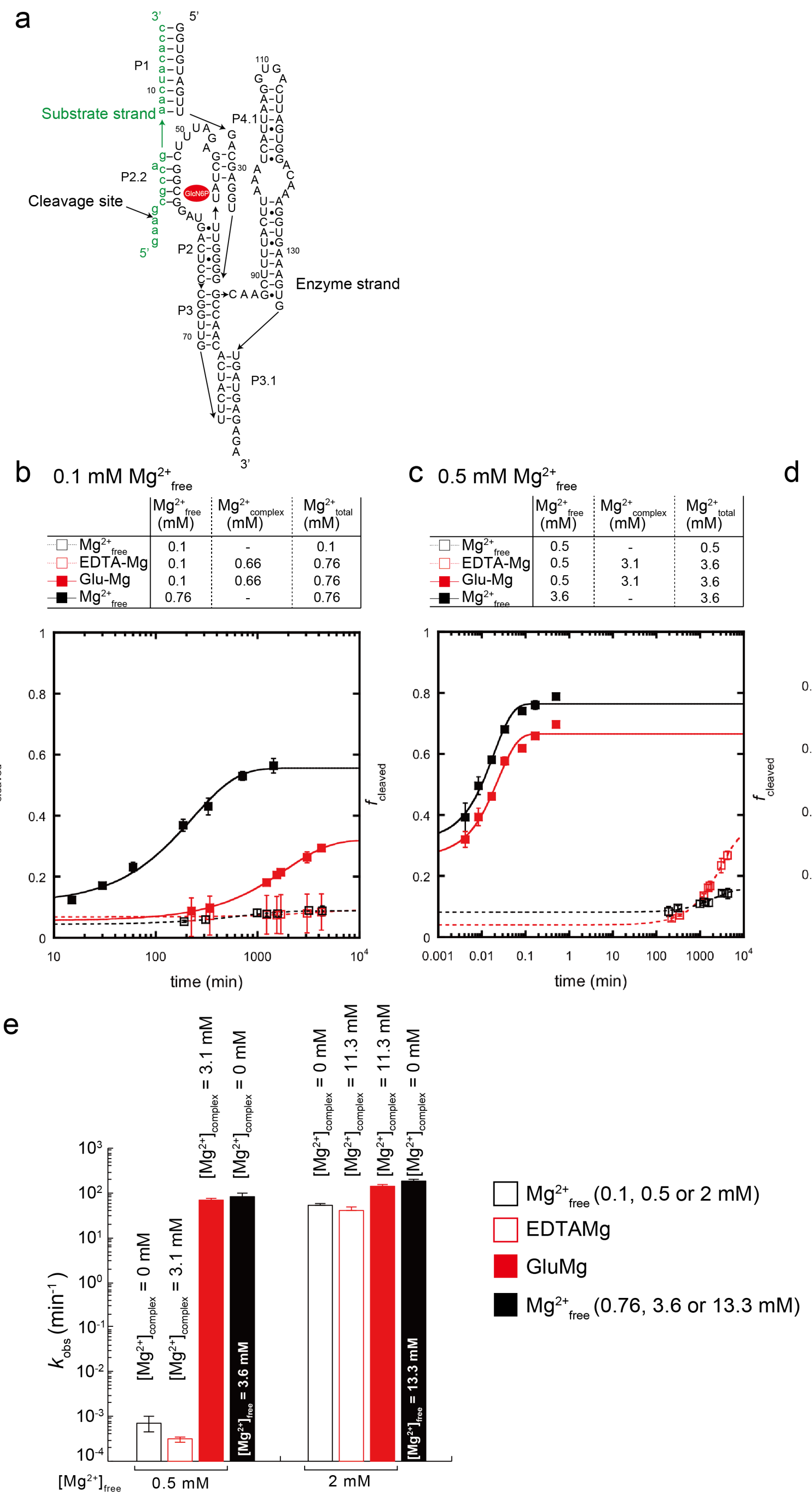
a
Substrate strand
P1
P2.2
P2
P3
P3.1
P4.1
Cleavage site
Enzyme strand
GlcN6P
b
0.1 mM Mg2+ free
Mg2+ free (mM) | Mg2+ complex (mM) | Mg2+ total (mM)
Mg2+ free 0.1 - 0.1
EDTA-Mg 0.1 0.66 0.76
Glu-Mg 0.1 0.66 0.76
Mg2+ free 0.76 - 0.76
c
0.5 mM Mg2+ free
Mg2+ free 0.5 - 0.5
EDTA-Mg 0.5 3.1 3.6
Glu-Mg 0.5 3.1 3.6
Mg2+ free 3.6 - 3.6
d
2 mM Mg2+ free
Mg2+ free 2.0 - 2.0
EDTA-Mg 2.0 11.3 13.3
Glu-Mg 2.0 11.3 13.3
Mg2+ free 13.3 - 13.3
f cleaved
time (min)
e
k obs (min-1)
[Mg2+]complex = 0 mM
[Mg2+]complex = 3.1 mM
[Mg2+]complex = 11.3 mM
[Mg2+]free = 3.6 mM
[Mg2+]free = 13.3 mM
[Mg2+]free
0.5 mM
2 mM
Mg2+ free (0.1, 0.5 or 2 mM)
EDTAMg
GluMg
Mg2+ free (0.76, 3.6 or 13.3 mM)

Supplementary Fig. 4 | Enzymatic activity of the *glmS* ribozyme is stimulated in both bacterial and eukaryotic cell mimic conditions. (a) The *glmS* ribozyme is prepared in two pieces, which are substrate strand (green) and enzyme strand (black). The *glmS* ribozyme binds glucosamine-6-phosphate (GlcN6P; red) as a cofactor that is required for catalysis[4]. The cleavage site is after nucleotide 3 in the substrate strand. (b) Self-cleaving reaction at 0.1 mM Mg^{2+}_{free} condition. Fraction ($f_{cleaved}$) vs time plot is shown. Black open squares show 0.1 mM Mg^{2+}_{total} for control, red open squares show 0.66 mM EDTA, red filled squares show 96 mM glutamate and black squares show 0.76 mM Mg^{2+}_{total}. (c) Self-cleaving reaction at 0.5 mM Mg^{2+}_{free} condition. (d) Self-cleaving reaction at 2 mM Mg^{2+}_{free} condition. Concentrations of binding donors for (c) are provided in Figure 1; For (b), binding donor concentrations are 96 mM and 0.66 mM for glutamate and EDTA. (e) k_{obs} values for the cleaving reaction at the magnesium conditions. The error bars mean S.D. (n=3). The symbols and colors are same with **b**, **c** and **d**. For 0.1 mM Mg^{2+}_{free}, reaction was barely detectable above background in the absence of chelated Mg^{2+}.

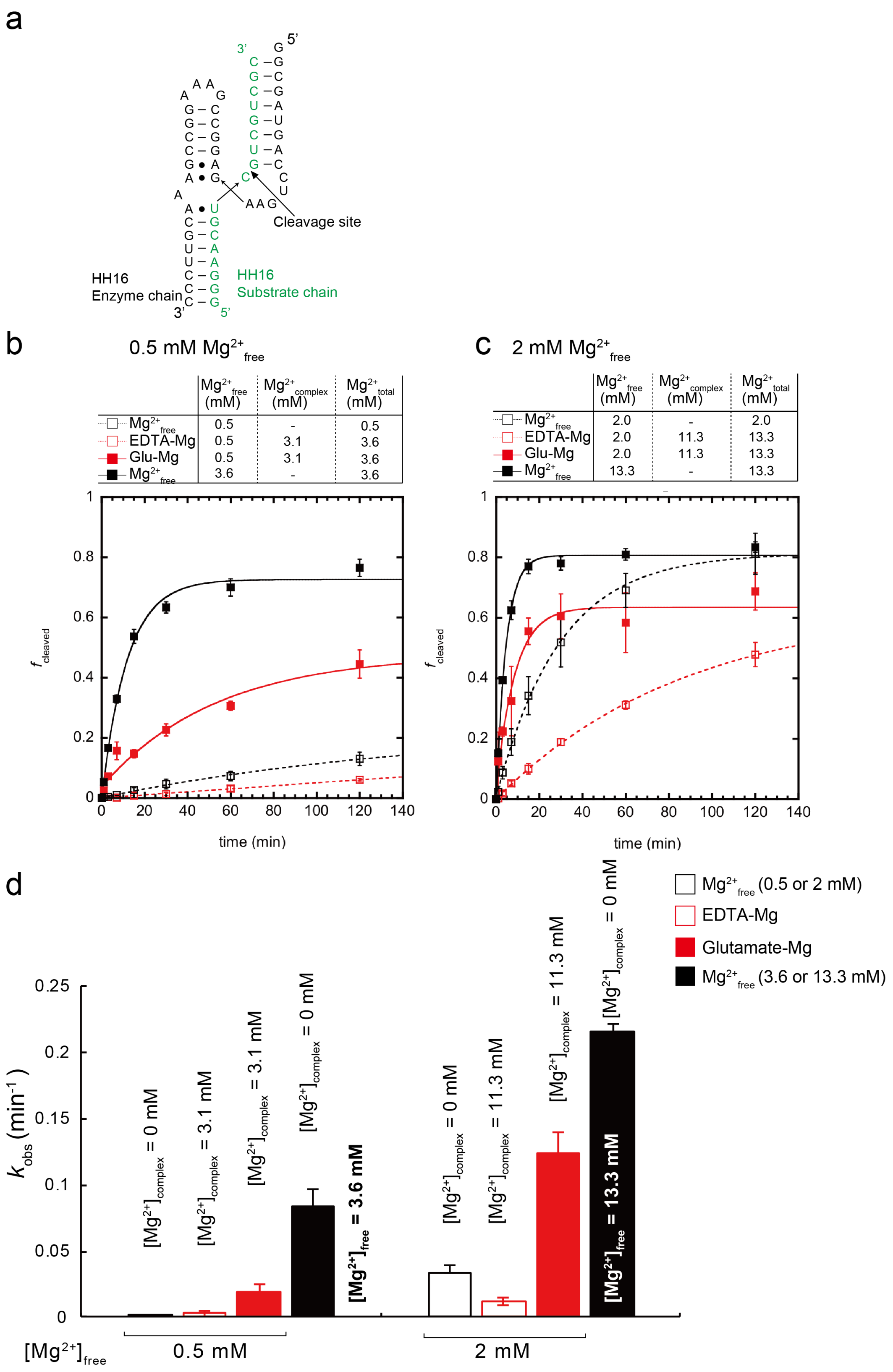
a
3'
5'
Cleavage site
HH16
Enzyme chain
HH16
Substrate chain
b
0.5 mM Mg2+ free
Mg2+ free (mM)
Mg2+ complex (mM)
Mg2+ total (mM)
Mg2+ free 0.5 - 0.5
EDTA-Mg 0.5 3.1 3.6
Glu-Mg 0.5 3.1 3.6
Mg2+ free 3.6 - 3.6
f cleaved
time (min)
c
2 mM Mg2+ free
Mg2+ free (mM)
Mg2+ complex (mM)
Mg2+ total (mM)
Mg2+ free 2.0 - 2.0
EDTA-Mg 2.0 11.3 13.3
Glu-Mg 2.0 11.3 13.3
Mg2+ free 13.3 - 13.3
f cleaved
time (min)
d
kobs (min-1)
[Mg2+]complex = 0 mM
[Mg2+]complex = 3.1 mM
[Mg2+]complex = 3.1 mM
[Mg2+]complex = 0 mM
[Mg2+]free = 3.6 mM
[Mg2+]complex = 0 mM
[Mg2+]complex = 11.3 mM
[Mg2+]complex = 11.3 mM
[Mg2+]complex = 0 mM
[Mg2+]free = 13.3 mM
[Mg2+]free
0.5 mM
2 mM
Mg2+ free (0.5 or 2 mM)
EDTA-Mg
Glutamate-Mg
Mg2+ free (3.6 or 13.3 mM)

Supplementary Fig. 5 | Enzymatic activity of the hammerhead ribozyme 16 is stimulated by weakly bound magnesium.

(a) The HH16 is prepared in two pieces which are substrate strand (green) and enzyme strand (black). The cleavage site is after position 9 in the substrate strand. (b) Self-cleaving reaction at 0.5 mM Mg^{2+}_{free} condition. Fraction ($f_{cleaved}$) vs time plot is shown. Black open squares show 0.5 mM Mg^{2+}_{total} for control, red open squares show 3.6 mM EDTA, red filled squares show 96 mM glutamate and black squares show 3.6 mM Mg^{2+}_{total}. (c) Self-cleaving reaction at 2 mM Mg^{2+}_{free} condition. Concentrations of binding donors for (c) are provided in Figure 1; For (b), binding donor concentrations are 96 mM and 0.66 mM for glutamate and EDTA. (d) k_{obs} values for the cleaving reaction at the various magnesium conditions. The error bars mean S.D. (n=3). The symbols and colors are same with **b** and **c**.

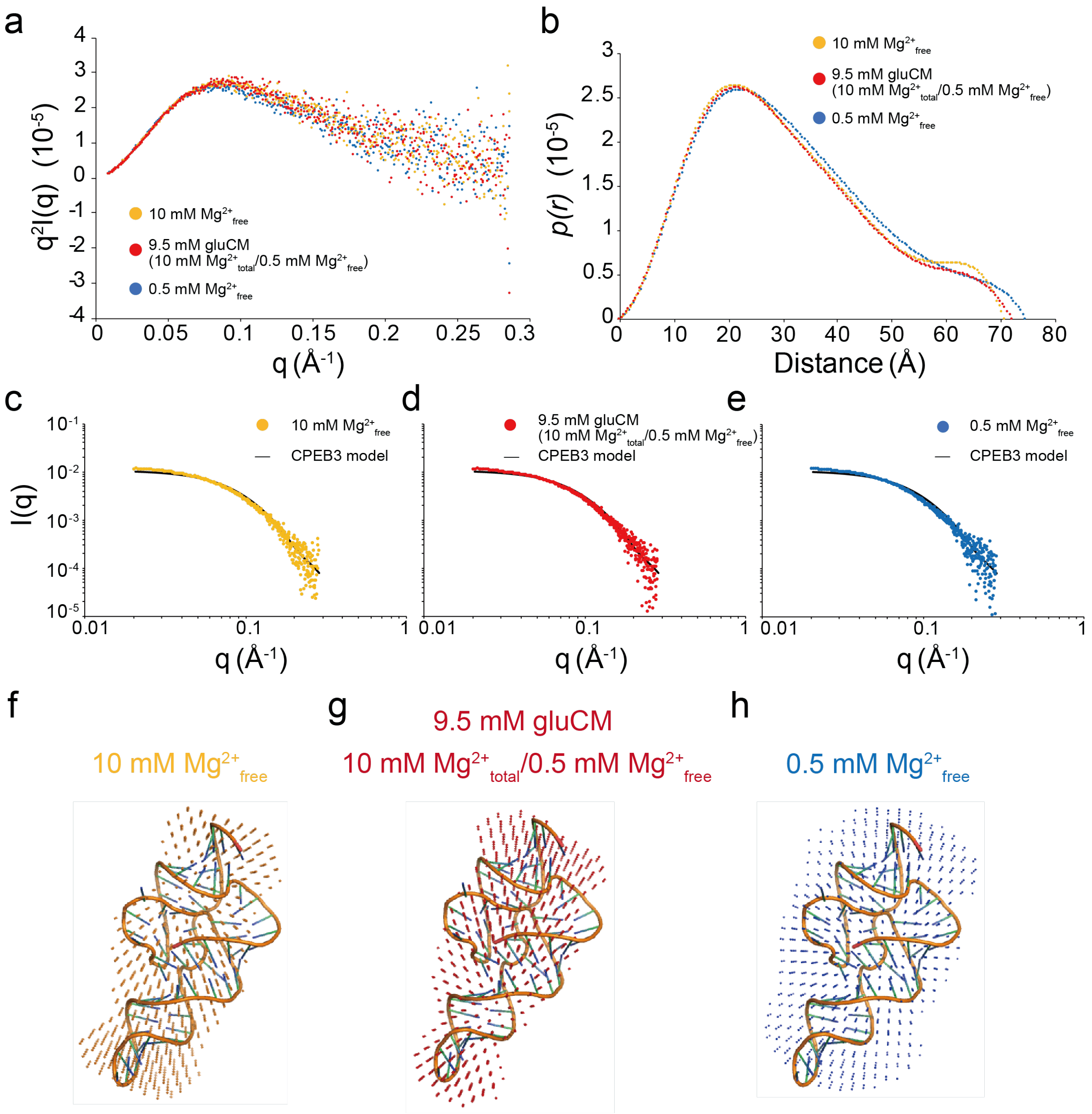

Supplementary Fig. 6 | Glutamate-chelated magnesium contributes to RNA compaction. SAXS experiments were carried out using CPEB3 ribozyme. (a, b) Dimensionless Kratky plot ($q^2I(q)$ vs q) and *p(r)* plot in 10 mM Mg^{2+}_{free} (yellow), 9.5 mM gluCM (red), and 0.5 mM Mg^{2+}_{free} (blue). (c, d, e) The experimental scattering curve in each condition is compared to the theoretical scattering curve which was created by FoXS software. (f, g, h) The crystal structure of CPEB3 ribozyme modeled from the crystal structure of HDV-ribozyme (3NKB) was aligned with the bead model created by DAMMIF using the scattering data in each condition.

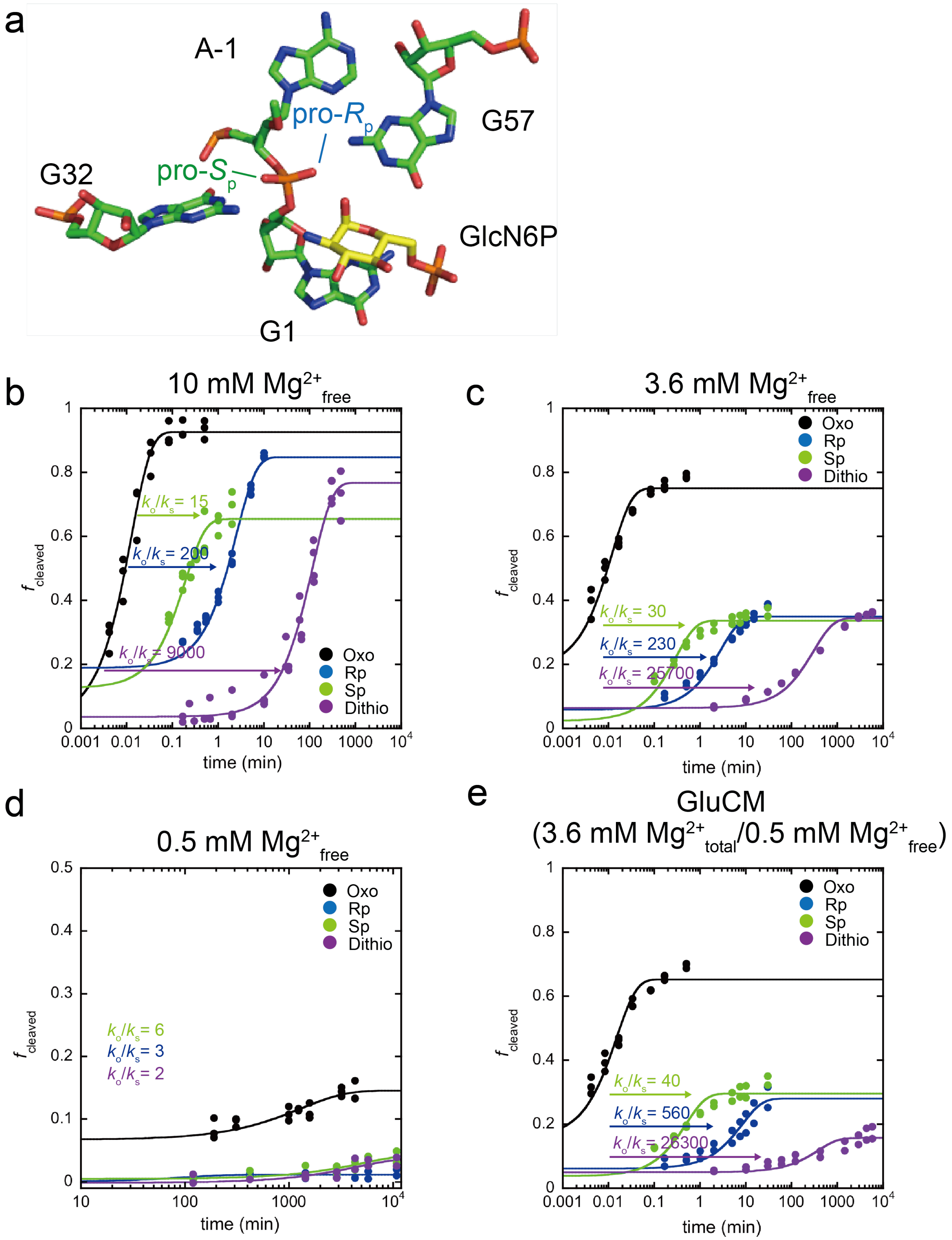

Supplementary Fig. 7 | *glmS* ribozyme shows similar thio effects in 0.5 mM Mg^{2+}_{free} and 3.1 mM gluCM as 3.6 mM Mg^{2+}_{free}.

(a) Close-up view of the catalytic center of the *glmS* ribozyme (PDB: 2NZ4). Sulfur-substitution positions at the non-bridging oxygen of pro-R_p (blue) and pro-S_p (green) in the scissile phosphate are shown. (b) Thio effect data in 10 mM Mg^{2+}_{free} condition for the R_p (blue), S_p (green), and dithio (purple) substrates compared with the oxo substrate (black). These data were obtained from reference.[4] (c, d) Thio effect data in 3.6 mM and 0.5 mM Mg^{2+}_{free}, respectively. (e) Thio effect in 3.1 mM gluCM (3.6 mM Mg^{2+}_{total}/0.5 mM Mg^{2+}_{free}) condition.

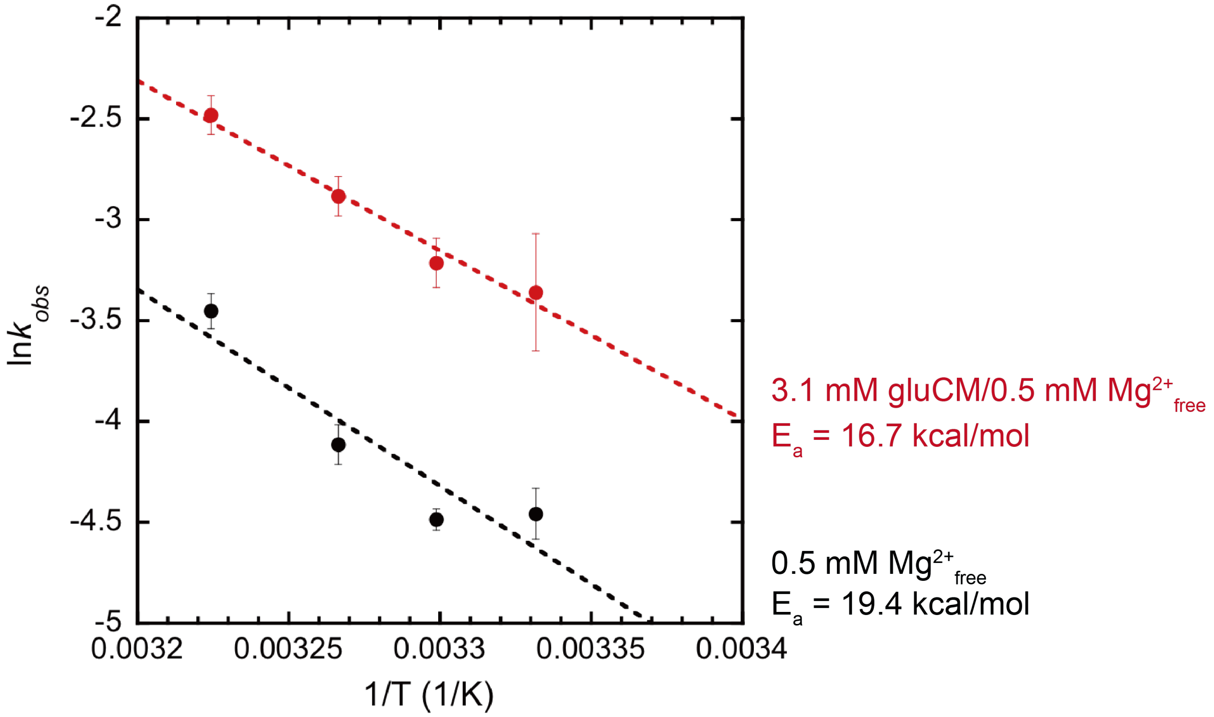

Supplementary Fig. 8 | Weakly chelated magnesium shows similar free-activation energy to unbound magnesium.

The CPEB3 activity was measured at different four temperatures (27, 30, 33, and 37°C) in either 3.1 mM gluCM/0.5 mM Mg^{2+}_{free} (red filled circles) or 0.5 mM Mg^{2+}_{free} (black filled circles). The free-activation energy (E_a) was calculated using Arrhenius Equation. The slops ($-E_a/R$) in 3.1 mM gluCM/0.5 mM Mg^{2+}_{free} or 0.5 mM Mg^{2+}_{free} were -8400 and -9800, respectively. The E_a in the gluCM condition is similar to that in the magnesium condition. The error bars mean S.D. (n=3).

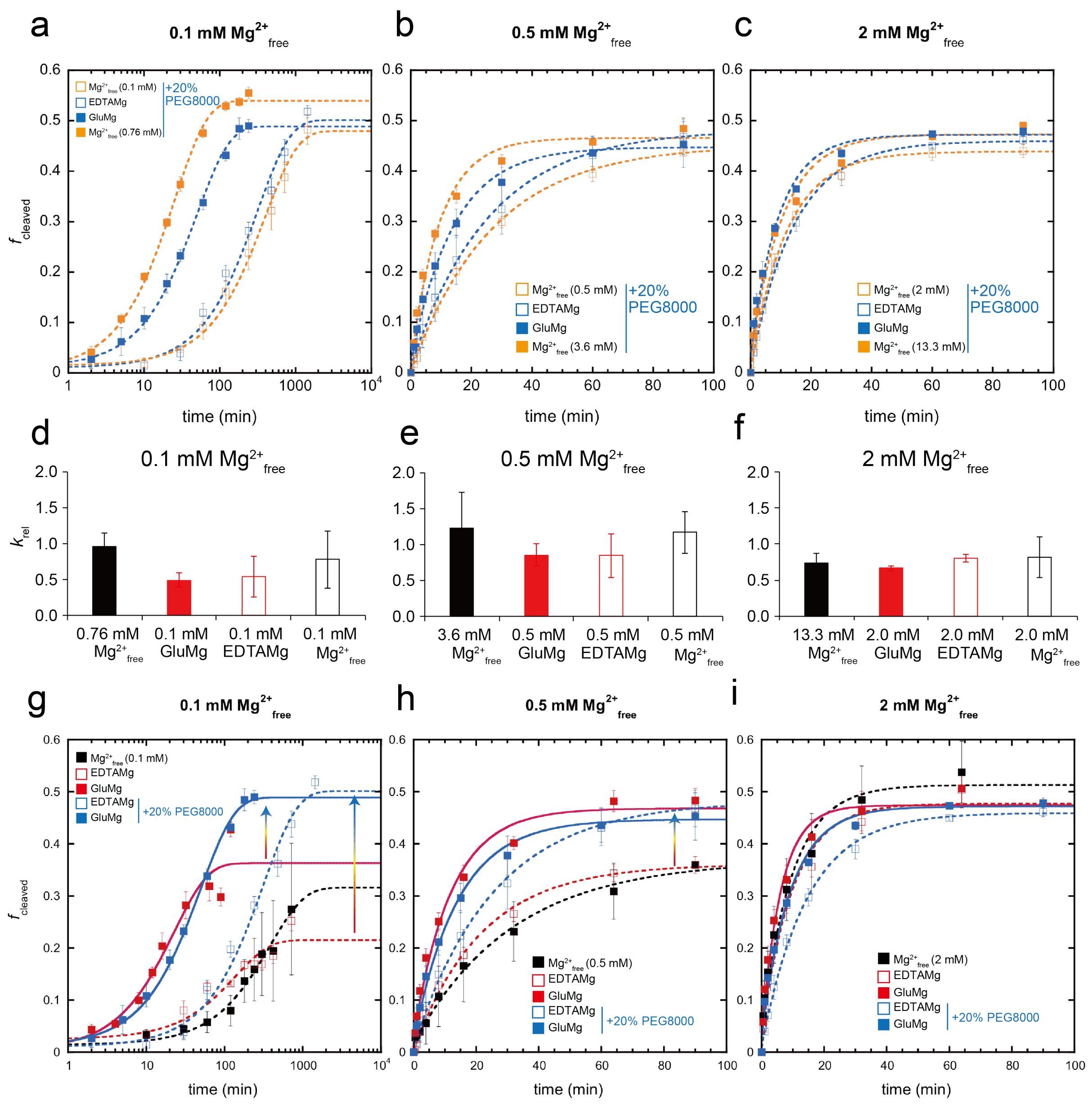

Supplementary Fig. 9 | Crowding conditions with chelated magnesium show greater amplitudes for RNA catalysis.

(a) The CPEB3 activity was tested in various chelated magnesium concentrations with 20% PEG8000. Orange opened squares show 0.1 mM Mg^{2+}_{total} for control, blue opened squares show 0.1 mM EDTACM, blue filled squares show 0.1 mM gluCM with 20% PEG8000, orange filled squares show 0.76 mM Mg^{2+}_{total}. (b, c) 0.5 mM and 2 mM Mg^{2+}_{free} concentrations were tested. The symbols and color are same with **a**. (d, e, f) k_{rel} values are shown. The k_{rel} values are calculated from the k_{obs} in aaCM plus PEG8000 divided the k_{obs} in aaCM. The error bars mean S.D. (n=3 or 4). (g, h, i) Comparison of amplitudes in the presence and absence of 20% PEG8000. Black squares show 0.1 mM Mg^{2+}_{total} for control, red opened squares show 0.1 mM EDTACM, red filled squares show 0.1 mM gluCM, blue opened squares show 0.1 mM EDTACM with 20% PEG8000, and blue filled squares shows 0.1 mM gluCM with 20% PEG8000. The error bars mean S.D. (n=3 or 4). Gradient arrows show stimulation of amplitude.

3. Supplementary Tables

Supplementary Table 1 | Estimated concentrations of cellular amino acids and magnesium complexes in *Escherichia coli*

$[Mg^{2+}]_{free}$	Amino acids	Cellular abundance (mM)[a]	K_D for magnesium (mM)[b]	Estimated magnesium complex in cells (mM)[c]		$f_{AA \cdot Mg^{2+}}$ [d]
2 mM (prok.)	L-glutamate	96.0	15.1	11.3		0.12
	L-aspartate	4.2	3.2	1.7		0.39
	L-glutamine	3.8	12.6	0.6		0.14
	L-alanine	2.6	11.0	0.4	Σ=14 mM	0.15
0.5 mM (euk.)	L-glutamate	96.0	15.1	3.1		0.03
	L-aspartate	4.2	3.2	0.6		0.14
	L-glutamine	3.8	12.6	0.2		0.04
	L-alanine	2.6	11.0	0.2	Σ=4.1 mM	0.04
0.01 mM (neg. ctrl.)	L-glutamate	96.0	15.1	0.064		0.001
	L-aspartate	4.2	3.2	0.013		0.003
	L-glutamine	3.8	12.6	0.003		0.001
	L-alanine	2.6	11.0	0.003	Σ=0.083 mM	0.001
	Magnesium	54	-	-		

[a]The cellular abundances of the amino acids were obtained from[2].
[b]The binding affinities of amino acids for magnesium were obtained from[5,6].
[c]Calculated according to equation 5. [d]Calculated according to equation 4.

Supplementary Table 2 | All melting profiles

Ribozyme name	Condition	$[Mg^{2+}]_{free}$ (mM)	T_M (°C)	ΔH (kcal/mol)	$\Delta\Delta G^a$ (kcal/mol)	Figure
drz-spur-3	0 mM glutamate	0	56.4 ± 0.2	-55.3 ± 0.1	0.0	SF3a
	9.6 mM glutamate	0	55.8 ± 0.3	-58.3 ± 1.1	0.6 ± 0.2	SF3a
	96 mM glutamate	0	58.4 ± 0.2	-63.4 ± 0.9	-1.9 ± 0.1	SF3a
drz-spur-3	0 mM additional KCl	0	55.8 ± 0.2	-52.6 ± 1.7	0.0	SF3b
	9.6 mM additional KCl	0	56.1 ± 0.0	-58.7 ± 1.0	0.3 ± 0.2	SF3b
	96 mM additional KCl	0	59.1 ± 0.1	-64.8 ± 1.0	-2.6 ± 0.2	SF3b
drz-spur-3	2 mM Mg^{2+}	2	65.1 ± 0.2	-105.1 ± 4.5	0.0	1a
	EDTCM	2	64.9 ± 0.1	-98.6 ± 1.0	0.4 ± 0.5	1a
	Aa_4CM	2	69.9 ± 0.1	-112.0 ± 0.7	-7.2 ± 0.1	1a
	GluCM	2	69.7 ± 0.2	-125.0 ± 4.0	-6.9 ± 0.3	1a
	13.3 mM Mg^{2+}	13.3	70.0 ± 0.1	-105.9 ± 6.1	-7.3 ± 0.1	1a
drz-spur-3	0.5 mM Mg^{2+}	0.5	61.1 ± 0.2	-90.9 ± 3.4	0.0	1b
	EDTACM	0.5	60.7 ± 0.1	-89.1 ± 1.3	0.6 ± 0.3	1b
	Aa_4CM	0.5	66.8 ± 0.1	-112.2 ± 4.4	-7.7 ± 0.1	1b
	GluCM	0.5	66.2 ± 0.1	-115.3 ± 1.5	-6.9 ± 0.1	1b
	3.6 mM Mg^{2+}	3.6	66.8 ± 0.1	-102.4 ± 3.3	-7.7 ± 0.3	1b
drz-spur-3	0 mM Mg^{2+}	0	56.7 ± 0.1	-62.5 ± 2.9	0.0	1c
	EDTACM	0.01	56.2 ± 0.3	-60.0 ± 2.2	0.6 ± 0.2	1c
	Aa_4CM	0.01	59.9 ± 0.1	-67.3 ± 0.8	-3.4 ± 0.1	1c
	GluCM	0.01	59.4 ± 0.1	-72.5 ± 0.3	-3.0 ± 0.1	1c
	0.076 mM Mg^{2+}	0.076	56.8 ± 0.1	-60.5 ± 5.8	-0.1 ± 0.1	1c
drz-spur-3	1.1 mM Mg^{2+}	1.1	63.4 ± 0.1	-89.8 ± 2.9	0.0	5
	EDTACM	0.0	60.9 ± 0.1	-58.9 ± 0.3	3.6 ± 0.2	5
	CitrateCM	0.05	59.2 ± 0.1	-70.5 ± 1.3	6.3 ± 0.3	5
	MalateCM	1.1	64.4 ± 0.2	-104.3 ± 1.8	-1.4 ± 0.3	5
	GluCM	1.1	66.1 ± 0.1	-106.3 ± 2.0	-3.6 ± 0.1	5
	3 mM Mg^{2+}	3.0	66.1 ± 0.1	-100.2 ± 0.2	-3.6 ± 0.1	5

[a] $\Delta\Delta G$ was calculated from eq10 and used the first ΔH in each grouping.

Supplementary Table 3| Kinetics parameters of CPEB3 ribozyme for self-cleaving reaction in several conditions

Condition	Reaction temperature (°C)	$[Mg^{2+}]_{free}$ (mM)	$[Mg^{2+}]_{complex}$ (mM)	$[Mg^{2+}]_{total}$ (mM)	PEG8000 (%)	k_{obs} (min^{-1})	Fold-stimulation
Mg^{2+}	37	0.1	0	0.1	0	0.003 ± 0.001	1.0 ± 0.4
EDTACM	37	0.1	0.66	0.76	0	0.007 ± 0.004	2.8 ± 1.8
GluCM	37	0.1	0.66	0.76	0	0.044 ± 0.009	17 ± 6
Mg^{2+}	37	0.76	0	0.76	0	0.041 ± 0.007	16 ± 5
Mg^{2+}	37	0.1	0	0.1	20	0.002 ± 0.001	0.8 ± 0.3
EDTACM	37	0.1	0.66	0.76	20	0.004 ± 0.001	1.5 ± 0.5
GluCM	37	0.1	0.66	0.76	20	0.020 ± 0.001	7.7 ± 2
Mg^{2+}	37	0.76	0	0.76	20	0.039 ± 0.001	15 ± 4
Mg^{2+}	37	0.5	0	0.5	0	0.032 ± 0.005	1.0 ± 0.2
EDTACM	37	0.5	3.1	3.6	0	0.046 ± 0.003	1.4 ± 0.2
GluCM	37	0.5	3.1	3.6	0	0.084 ± 0.012	2.6 ± 0.5
Mg^{2+}	37	3.6	0	3.6	0	0.091 ± 0.041	2.9 ± 1.4
Mg^{2+}	37	0.5	0	0.5	20	0.037 ± 0.002	1.2 ± 0.2
EDTACM	37	0.5	3.1	3.6	20	0.039 ± 0.010	1.2 ± 0.4
GluCM	37	0.5	3.1	3.6	20	0.071 ± 0.003	2.3 ± 0.3
Mg^{2+}	37	3.6	0	3.6	20	0.103 ± 0.001	3.2 ± 0.5
Mg^{2+}	37	2	0	2	0	0.10 ± 0.03	1.0 ± 0.4
EDTACM	37	2	11.3	13.3	0	0.09 ± 0.01*	0.9 ± 0.2
GluCM	37	2	11.3	13.3	0	0.16 ± 0.01*	1.6 ± 0.4
Mg^{2+}	37	13.3	0	13.3	0	0.13 ± 0.02	1.2 ± 0.4
Mg^{2+}	37	2	0	2	20	0.084 ± 0.011	0.8 ± 0.2
EDTACM	37	2	11.3	13.3	20	0.069 ± 0.007	0.7 ± 0.2
GluCM	37	2	11.3	13.3	20	0.11 ± 0.008	1.0 ± 0.3
Mg^{2+}	37	13.3	0	13.3	20	0.094 ± 0.003	0.9 ± 0.2
Mg^{2+}	33	0.5	0	0.5	0	0.016 ± 0.002	1.0 ± 0.1
GluCM	33	0.5	3.1	3.6	0	0.056 ± 0.005	3.4 ± 0.5
Mg^{2+}	30	0.5	0	0.5	0	0.011 ± 0.001	1.0 ± 0.1
GluCM	30	0.5	3.1	3.6	0	0.040 ± 0.005	3.6 ± 0.5
Mg^{2+}	27	0.5	0	0.5	0	0.012 ± 0.001	1.0 ± 0.2
GluCM	27	0.5	3.1	3.6	0	0.035 ± 0.010	3.0 ± 1.0

Error means S.D. (n=3 or 4).

* minimum error was estimated at 0.01 min^{-1} due to various sources of systematic error.

Supplementary Table 4| Kinetics parameters of *glmS* ribozyme for self-cleaving reaction in several conditions

Condition	$[Mg^{2+}]_{free}$ (mM)	$[Mg^{2+}]_{complex}$ (mM)	$[Mg^{2+}]_{total}$ (mM)	k_{obs} (min^{-1})	Fold-stimulation
Mg^{2+}	0.1	0	0.1	*	*
EDTACM	0.1	0.66	0.76	*	*
GluCM	0.1	0.66	0.76	0.0006	*
Mg^{2+}	0.76	0	0.76	0.006	*
Mg^{2+}	0.5	0	0.5	0.0007 ± 0.0003	1.0 ± 0.4
EDTACM	0.5	3.1	3.6	0.0003 ± 0.00003	0.5 ± 0.1
GluCM	0.5	3.1	3.6	67 ± 9	$(1.1 \pm 0.2) \times 10^5$
Mg^{2+}	3.6	0	3.6	86 ± 11	$(1.3 \pm 0.24) \times 10^5$
Mg^{2+}	2	0	2	52 ± 9	1.0 ± 0.2
EDTACM	2	11.3	13.3	41 ± 6	0.8 ± 0.1
GluCM	2	11.3	13.3	145 ± 12	2.8 ± 0.3
Mg^{2+}	13.3	0	13.3	185 ± 16	3.6 ± 0.4

Error means S.D. (n=3).
* difficult to estimate due to slow reaction.

Supplementary Table 5| Kinetics parameters of hammerhead 16 for self-cleaving reaction in several conditions

Condition	$[Mg^{2+}]_{free}$ (mM)	$[Mg^{2+}]_{complex}$ (mM)	$[Mg^{2+}]_{total}$ (mM)	k_{obs} (min^{-1})	Fold-stimulation
Mg^{2+}	0.5	0	0.5	0.001 ± 0.0002	1.0 ± 0.2
EDTACM	0.5	3.1	3.6	0.003 ± 0.002	2.6 ± 1.7
GluCM	0.5	3.1	3.6	0.020 ± 0.006	17 ± 6
Mg^{2+}	3.6	0	3.6	0.084 ± 0.013	74 ± 15
Mg^{2+}	2	0	2	0.034 ± 0.01	1.0 ± 0.2
EDTACM	2	11.3	13.3	0.012 ± 0.003	0.3 ± 0.1
GluCM	2	11.3	13.3	0.12 ± 0.02	3.6 ± 0.7
Mg^{2+}	13.3	0	13.3	0.22 ± 0.01	6.3 ± 1.0

Error means S.D. (n=3).

Supplementary Table 6 | SAXS determined parameters in various conditions

Condition	$[Mg^{2+}]_{free}$ (mM)	$[Mg^{2+}]_{complex}$ (mM)	$R_{g\ (Guinier)}$	D_{max} (Å)	Molecular Weight (g/mol)
10 mM Mg^{2+}_{free}	10	0	25.7	71	27.8
GluCM	0.5	9.5	26.3	72	27.3
0.5 mM Mg^{2+}_{free}	0.5	0	27.0	75	28.8

Supplementary Table 7 | Thio effect for self-cleaving reaction in several conditions

Condition	$[Mg^{2+}]_{free}$ (mM)	$[Mg^{2+}]_{complex}$ (mM)	$[Mg^{2+}]_{total}$ (mM)	Substrate	k_{obs} (min^{-1})	k_O/k_S	Ref.
10 mM Mg^{2+}_{free}	10	0	10	oxo	50 ± 3	-	4
	10	0	10	R_p	0.40 ± 0.05	200 ± 50	4
	10	0	10	S_p	5 ± 1	15 ± 5	4
	10	0	10	dithio	0.009 ± 0.001	9000 ± 3000	4
3.6 mM Mg^{2+}_{free}	3.6	0	3.6	oxo	86 ± 11	-	-
	3.6	0	3.6	R_p	0.37 ± 0.06	230 ± 40	-
	3.6	0	3.6	S_p	3.4 ± 0.5	30 ± 10	-
	3.6	0	3.6	dithio	0.003 ± 0.002	25700 ± 3600	-
0.5 mM Mg^{2+}_{free}	0.5	0	0.5	oxo	0.00073 ± 0.00028	-	-
	0.5	0	0.5	R_p	0.0002 ± 0.0001	3 ± 1	-
	0.5	0	0.5	S_p	0.00018 ± 0.00015	6 ± 3	-
	0.5	0	0.5	dithio	0.00052 ± 0.00041	2 ± 1	-
0.5 mM gluCM	0.5	3.1	3.6	oxo	67 ± 9	-	-
	0.5	3.1	3.6	R_p	0.12 ± 0.01	560 ± 50	-
	0.5	3.1	3.6	S_p	2.2 ± 1.2	40 ± 10	-
	0.5	3.1	3.6	dithio	0.0026 ± 0.0002	26300 ± 2900	-

Error means S.D. (n=3 for oxo substrates and n=4 for the other substrates)

4. References

1. Outten, C.E. & O'Halloran, T.V. Femtomolar sensitivity of metalloregulatory proteins controlling zinc homeostasis. *Science* **292**, 2488-92 (2001).
2. Bar-Even, A., Noor, E., Flamholz, A., Buescher, J.M. & Milo, R. Hydrophobicity and charge shape cellular metabolite concentrations. *PLoS Comput. Biol.* **7**, e1002166 (2011).
3. Bennett, B.D. *et al*. Absolute metabolite concentrations and implied enzyme active site occupancy in *Escherichia coli*. *Nat. Chem. Biol.* **5**, 593-9 (2009).
4. Bingaman, J.L. *et al*. The GlcN6P cofactor plays multiple catalytic roles in the *glmS* ribozyme. *Nat. Chem. Biol.* **13**, 439-445 (2017).
5. Martell, A.E. & Smith, R.M. *CRITICAL STABILITY CONSTANTS*, 469 (Plenum Press, New York, 1974).
6. Martell, A.E. & Smith, R.M. *CRITICAL STABILITY CONSTANTS Second Supplement*, (Springer, 1989).
