# Supplementary material for: Cellular conditions of weakly chelated magnesium ions strongly promote RNA stability and catalysis
Source: Nat Commun. 2018 Jun 1;9:2149. doi: 10.1038/s41467-018-04415-1 (PMC5984629; doi:10.1038/s41467-018-04415-1)
Supplement: Supplementary file 3 — Description of Additional Supplementary Files [file 41467_2018_4415_MOESM3_ESM.pdf]

Description of Additional Supplementary Files:

File Name: Supplementary Dataset 1
Description: This dataset describes all of our experimental reaction conditions.
